# Willingness to adopt personal biosecurity strategies on thoroughbred breeding farms: Findings from a multi-site pilot study in Australia's Hunter Valley

**DOI:** 10.3389/fvets.2022.1017452

**Published:** 2022-12-15

**Authors:** Kirrilly Thompson, Joanne Taylor, Diana Mendez, Catherine Chicken, Joan Carrick, David N. Durrheim

**Affiliations:** ^1^Hunter New England Local Health District, Wallsend, NSW, Australia; ^2^College of Health, Medicine and Wellbeing, University of Newcastle, Callaghan, NSW, Australia; ^3^Australian Institute of Tropical Health and Medicine, James Cook University, Townsville, QLD, Australia; ^4^Consultant Veterinarian, Scone Equine Hospital, Scone, NSW, Australia; ^5^Equine Specialist Consulting, Scone, NSW, Australia

**Keywords:** equine, thoroughbred, behavior change, biosecurity, infection control, zoonotic disease, personal biosecurity, Transtheoretical

## Abstract

There are almost 9,500 full-time employees in Australia's thoroughbred horse breeding industry. During foaling, they can be exposed to bodily fluids and mucous membranes which may present risks for zoonotic disease. These risks can be mitigated through personal biosecurity strategies. The aim of this study was to identify which personal biosecurity strategies were more or less likely to be adopted by workers. Seventeen participants representing 14 thoroughbred breeding farms and three equine veterinary practices in Australia's largest thoroughbred breeding region trialed up to 16 stakeholder-nominated personal biosecurity strategies over the 2021 foaling season. The strategies encompassed personal protective equipment (PPE), zoonotic disease awareness, policies and protocols, supportive environments, and leadership. Strategy adoption was monitored through three repeated self-audit surveys designed around the Transtheoretical Model of change (TTM) and findings were reviewed in exit interviews. For all survey waves in aggregate, 13 strategies were practiced by at least 50.0% of participants. Participants were most likely to use a ready-made foaling box (98.0%), communicate the message that PPE usage is a personal responsibility (94.1%) and use ready-made PPE kits (88.2%). However, 31.4% had no intention of doing practice sessions and/or dummy runs for PPE use and 27.5% had no intention of using a buddy system on farm/practice to check use of PPE. Whilst these rates indicate workers' willingness to adopt and maintain personal biosecurity strategies, they also indicate capacity for more practices to be implemented more often. Overall, the findings highlight the need for personal biosecurity interventions to be sensitive to the demands of the annual thoroughbred breeding calendar, the size of the breeding operation and the availability of skilled staff.

## Introduction

Foaling season is the busiest and arguably most stressful time of the year for thoroughbred breeding farms. The unpredictability of foaling, the financial value of pedigree horses and high emotional investment add considerable pressure to the workload of reproductive staff who often work extended shifts to ensure the health and safety of mares and foals. With a gestation period averaging 344 days (1), abortions, stillbirths and the death of neonates represent a significant loss of time and money. When available, additional staff are recruited to meet the demands of the foaling season. Mares close to foaling are often observed around the clock (by night and day watch teams), with neonates being watched closely for days to weeks after birth for signs of any ill health. However, as workload and intensity increases, so too does the likelihood of fatigue-related impairment of decision-making. Moreover, as an example of “care work”, reproductive staff may prioritize the health of mares and foals over their own (i.e., patient safety), as has been noted in human healthcare and nursing (2). Whilst horse-related safety for equestrians and veterinarians is typically discussed in relation to physical accident and injury (3, 4), there are also risks of infection from zoonotic disease pathogens ranging from skin infections such as ringworm, to potentially fatal systemic infections, such as Hendra Virus (HeV) (5). In fact, one systematic review identified 56 zoonotic pathogens found in horses, of which 42 can be transmitted directly to humans and 14 *via* vectors (6).

Foaling and foal-handling are activities where humans can be exposed to various bodily materials, waste matter and other organic materials creating pathways for the transmission of zoonotic diseases, such as *salmonella* (7), *cryptosporidium parvum* (8, 9), *leptospirosis* (5) and *clostridioides* (10). For example, staff and student investigations of birth membranes at an Australian regional veterinary school in 2014 was linked to five human infections with *Chlamydia psittaci*, presenting with pneumonic symptoms (11, 12). Other forms of human exposure to *C. psittaci* related to foaling include uterine lavage, clean-up of aborted materials, foal resuscitation and direct contact with reproductive fluids or materials (13). A determination of the prevalence of *C. psittaci* infection amongst reproductive workers in the thoroughbred breeding industry suggested that transmission risk from horses to humans is probably low (13, 14). Still, there is a need to maintain strict hand hygiene and increase the use of personal protective equipment (PPE) such as gloves, coveralls, face masks and P2 respirator masks for known zoonotic diseases transmitted by the airborne route (15). Moreover, to protect themselves from emerging infectious zoonotic diseases, reproductive workers in the thoroughbred industry are advised to make continual risk assessments and take recommended precautions including wearing PPE. Not only are these personal biosecurity strategies important for protecting human health, they are also likely to contain the transmission of contagious diseases to other horses and thereby prevent further equine transmission or disease clusters on breeding farms. To that end, this study has been undertaken within a One Health framework that recognizes the interdependence of human and animal health (16). One Health is particularly salient for the thoroughbred industry where humans and horses interact in shared social and ecological contexts.

Following previous research (17) and the Australian Veterinary Association (15), the term “personal biosecurity” (PB) is used in this article to refer to all practices believed to reduce the risk of personal infection or transmission of zoonotic infection, including non-technical, social and behavioral interventions as well as PPE. Given the present study's focus on prevention, this terminology is preferred over the alternative, “infection control” employed in related research (18–20).

In Australia, research into horse-related biosecurity practices has mostly been in response to two sentinel events: HeV, first identified in 1994, and equine influenza (EI), of which there was an outbreak in 2007. The aforementioned 2014 outbreak of *C. psittaci* could be considered a third sentinel event, albeit at a reduced scale relative to EI and consequence relative to HeV. Research following these events has documented the barriers and enablers to personal biosecurity for workers in the horse industry as well as participants in recreational horse riding (17, 21–27). Findings have provided an in-depth understanding of problematic behaviors such as horse owner hesitancy to vaccinate horses against HeV (28), and proposed related interventions such as improving veterinarians' skills in risk communication (29). To our knowledge, the findings of this largely descriptive body of research have not been translated into field-based interventions designed to reduce the risk of zoonotic disease infection and transmission, as was the aim of the present study. In particular, the present study sought to investigate:

1. Which zoonoses were most relevant to reproductive workers in the thoroughbred breeding industry,
2. Which strategies were least likely to be adopted,
3. Which strategies were already being done to improve on-farm and personal biosecurity,
4. Which strategies were most likely to be considered or adopted, and
5. Which strategies were most likely to be discontinued?

This study also provided an opportunity to comment on the usefulness of applying the Transtheoretical Model of behavior change to the adoption of zoonotic disease prevention strategies.

## Materials and methods

### Site and timing

A two-phase multi-site study was undertaken in the Hunter Valley. With over 200 farms (30), the Hunter Valley in New South Wales is Australia's thoroughbred breeding capital (31) and the world's second largest concentration of thoroughbred breeding farms. One in two Australian racehorses are born there each year (30), contributing an estimated A$655 million annually to an industry valued at 1.2 billion (32). There are almost 9,500 full-time employees in Australia's thoroughbred breeding industry and almost 3,000 work in New South Wales for around 2,200 breeders (32). As noted above in relation to One Health, the health of these thousands of workers and horses is mutually inclusive and dependent to a large degree on personal biosecurity practices.

Data were collected between September 2021 and April 2022, concurrent with the foaling season, which in the southern hemisphere occurs predominantly from August to November, although some foals are also born in July and December. The 2021 foaling season coincided with the ongoing COVID-19 pandemic.

### Recruitment

Consistent with the demands of a study involving multiple points of contact and forms of participation during a stressful period in the thoroughbred industry calendar, the sample was not intended to be representative. Following a convenience sampling strategy, invitations to participate were sent to 32 industry workers across 21 organizations (18 breeding stud farms and three veterinary practices/businesses), who were known to members of the research team and who had previously shown interest in the study. Follow up calls were made by the two industry champions/co-researchers in the research team (Chicken and Carrick). Upon receipt of a signed consent form, participants were sent a link to the online survey. Two participants from stud farms withdrew after providing a signed consent, due to time constraints. The final seventeen participants and their organization are described below.

### Data collection

Data were collected in two stages. Stage One involved a self-audit survey of the adoption or otherwise of 16 personal biosecurity strategies. To capture changes in adoption behavior over the 2021–2022 foaling season, three self-audit survey waves were administered (identified in this article as “W1”, “W2,” or “W3”). Stage Two involved semi-structured exit interviews designed to contextualize and expand on survey data. Together, the two stages provided information about *what* changes were or were not made over time, and *why* or why not.

#### Stage 1: Self-audit surveys

Self-audit surveys were designed to monitor the adoption of strategies that had been previously proposed by industry participants in collaboration with researchers during a stakeholder workshop held in Scone, New South Wales (NSW) in June 2021. Thirty-five stakeholders (veterinarians and farm managers) from the thoroughbred breeding industry attended that workshop (representing eleven breeding farms and eight veterinary businesses). They were presented with findings from a 2018 interview-based study that had been undertaken to identify the barriers and enablers of personal biosecurity practices (17), which had been summarized according to the following five themes:

1. Greater awareness of current and emerging infectious risks promotes use of PPE
2. Currently available PPE is not comfortable, practical or well-suited to equine reproductive work in Australia's hot climate
3. Creating supportive environments for personal biosecurity reduces risk of exposure to infectious materials
4. Strong leadership is required to implement sustainable change in workplace culture and practices; and
5. Policy and economic factors play an important role in adopting biosecurity and PB measures in the workplace.

At the 2021 stakeholder workshop, researchers and industry co-researchers moderated five small group discussions, each focused on one theme. Attendees self-selected the group in which they participated. Groups were guided to identify practical strategies that in their opinion would address the enablers or barriers relevant to their theme and that could be easily implemented to improve the personal biosecurity of workers. That exercise yielded 16 strategies that formed the basis of - and were translated through, the present study.

The adoption of strategies was monitored over three self-audit surveys. W1 self-audit surveys were completed from 6 to 30 September, 2021; W2 self-audit surveys were completed between 26 October and 16 November, 2021; and W3 self-audit surveys were completed from 3 December 2021 to 1 Feb 2022. Survey data were collected and managed using REDCap electronic data capture tools hosted at Hunter New England Local Health District.^1^

The overall purpose of the repeated self-audit surveys was to monitor participants' willingness to adopt the 16 stakeholder-nominated strategies over the foaling season, and to capture any additional strategies identified by participants. In this article, strategies are referred to as “Sx,” as shown in Table 1. In the self-audit survey tool, strategies were grouped under the same five themes identified in previous research (17). The first self-audit survey wave (W1) included an additional question asking participants to state what, in their opinion, were the top three zoonoses about which staff should be aware during routine equine reproduction work.

**Table 1:** Strategies included in the self-audit tool to improve on-farm and personal biosecurity practices on thoroughbred breeding farms/veterinary practices.

**Theme**	**Strategy number**	**Strategy wording, verbatim from self-audit survey tool**
Encouraging appropriate personal protective equipment (PPE) use	S1	Communicating the message that PPE usage is a personal responsibility that needs to be self-disciplined/motivated
	S2	Doing practice sessions and/or dummy runs for PPE use
	S3	Using a buddy system on farm/practice to check use of PPE (ie. when someone else watches you (or reminds you to) put on your PPE to check for any errors, missed steps or incorrect order. More information is available at the HTBA website)
	S4	Using the “when/then” technique to change behaviors (i.e., when you decide to anchor a new behavior on an existing behavior. e.g., “when I look at a placenta, then I wear mask, gloves and glasses.” More information is available at the HTBA website, with a template to personalize your own “when/then” technique)
Greater awareness of current and emerging zoonoses	S5	Making sure messaging about zoonotic disease risk is simple but serious
	S6	Sharing at least one personal story from someone with first or second hand experience of a zoonotic disease with staff on my farm/practice
Policies and protocols	S7	Updating staff induction and training materials regularly, to encourage on-farm and personal biosecurity
	S8	Having response protocols clearly visible in areas where people may be exposed to bodily fluids/mucous membranes of horses
Creating supportive environments	S9	Using rewards, humor and incentives to encourage on-farm and personal biosecurity
	S10	Displaying signage about on-farm and personal biosecurity with simple but serious key messages
	S11	Delegating responsibility for restocking and arranging biosecurity equipment in vehicles
	S12	Using a ready-made “foaling box”
	S13	Using ready-made, complete, labeled PPE kits
Strong leadership	S14	Holding at least one toolbox meeting (ie. a group meeting with stud teams to discuss zoonotic risks and mitigation strategies)
	S15	Regular refreshers about on-farm and personal biosecurity to mitigate zoonotic infectious disease
	S16	Implementing strategies to support staff well-being related to zoonotic infections disease
Something else?	N/A -multiple	In addition to the strategies already listed in this survey, you might be doing or thinking about something else to address the risk of zoonotic disease. If this is the case, please use this space to describe the strategy being used at your stud farm/practice

Based on a longstanding history of application in various public health contexts, the Trans-theoretical Model of behavior change (TTM) (33, 34) was selected as a suitable theoretical framework for informing the design and interpretation of the self-audit survey waves. The TTM is based on the theory that behavior change is neither simple nor binary. Rather, multiple and cyclic stages of change are involved in adopting new or relinquishing old behaviors. The TTM proposes five stages of change through which people progress, regress and cycle as they change their behaviors: pre-contemplation, contemplation, preparation, action and maintenance, which is quantified as change having been maintained for at least 6 months. Relapse and recycling through stages is common, especially in relation to addictive behaviors (35).

To determine participants' willingness to adopt the 16 personal biosecurity strategies, the survey tool used in the present study included response options that were closely aligned with the TTM stages of change. For each of the personal biosecurity strategies, participants were provided with statements reflecting the TTM stages of change and asked to select which statement most accurately reflected behavior at their thoroughbred farm/veterinary business at the time of survey completion. Action and maintenance stages were combined, because the focus on the 3-month 2021 foaling season was less than the 6-month period traditionally defining the TTM maintenance stage. As self-audit surveys were intended to be completed during the foaling season, provision was not made for a “not applicable” response option.

As shown in Table 2, the wording of responses differed between W1 and subsequent waves. W1 had an additional “baseline” statement “we already do this and we started before June 2021.” This made it possible to distinguish between strategies that had already been adopted prior to the workshop, and those that were adopted following the stakeholder workshop, and might therefore be reasonably related to attendance. The decision to include this additional statement in W1 necessitated slightly different wording to the “action” statements between W1 and W2-3 (see Table 2), but which still capture the qualities of the action stage of change.

**Table 2:** Response options provided in the surveys, showing differences between the first and subsequent survey waves (emboldened), their association with a Transtheoretical stage of change and their arrangement into four analytic categories.

**Transtheoretical stage of change:**		**Baseline**	**Pre-contemplation**	**Contemplation**	**Preparation**	**Action/ maintenance**	**Relapse**
Adoption statements based on TTM stages of change:	W1	**We already do this - and we started before June 2021**	We've no intention of doing this	We're thinking about doing this	We've decided to do this, but haven't started yet	**We've started doing this (after June 1)**	We were doing this, but we stopped
	W2-3	n/a	We've no intention of doing this	We're thinking about doing this	We've decided to do this, but haven't started yet	**We're still doing this**	We were doing this, but we stopped
Analytic category:		Doing	No intention	Considering		Doing	Stopped

In the presentation of findings throughout this article, the TTM-inspired stages have been operationalized into the following four categories: (1) doing (includes “already doing,” “started doing” and “still doing”), (2) considering (includes “thinking about doing” and “decided to do but not yet started”), (3) stopped, and (4) no intention. Survey responses are presented collectively per wave (i.e., not for individual participants).

A link to the W1 survey was sent by email within 1–3 days of receiving consent. Invitations for subsequent waves were emailed ~30 days after completion of the preceding Wave. However, a technical issue with W2-3 increased this period by ~10 days for some participants. Reminders were made for all outstanding surveys by a combination of email, SMS and phone call. This variety of forms of contact was necessary as participants were frequently engaged in work outside of standard hours and in outdoor environments. Participants were given the option to complete the survey via a personalized link, over the phone or on a hardcopy of the survey. Eight surveys were completed in hardcopy and entered online manually by a researcher. Three surveys were completed over the phone with the researcher entering responses into the online survey. Delayed responses meant that the anticipated 3-month period of data collection lasted approximately 5 months.

#### Stage 2: Exit interviews

At the conclusion of the self-audit surveys, participants were invited to take part in face-to-face semi-structured exit interviews. Thirteen exit interviews were conducted between 15 March and 17 April, 2022. The exit interview guide was designed to elicit the broader context for individual survey responses, and to gain feedback about the research process overall (the latter of which is not the subject of the present article). Given that COVID-19-related travel restrictions had prevented the lead researcher from meeting participants prior to data collection, exit interviews also provided an opportunity to build rapport to facilitate the translation of findings and/or engagement with future research.

Exit interviews were held on location at studs, farms or veterinary practices in the Hunter Valley, with the exception of one in a local café and one via Zoom online conferencing. Interviewees were presented with a hardcopy of their responses to the three self-audit surveys. Differences between waves had been marked with a highlighter and a numerical indication of how much change had been made and in which direction (e.g., “+1” if someone had moved from contemplation to preparation or “−3” if they had moved from action to pre-contemplation). In addition, any instance of “no intention” was highlighted. This provided a focus around which to initiate a guided conversation about the reasons for change, the barriers and enablers to forward movement through the stages and reasons why some strategies had been stopped or never intended. Interviewees were also asked to reflect on what worked well, what was difficult, what they would have done differently and if they would do anything differently during the next foaling season.

The study protocol was approved by the Hunter New England Human Research Ethics Committee (Protocol 2019/ETH01070).

### Analysis

Survey data were analyzed with descriptive statistics, relative to the total sample. There was only one incident of missing data (P15, W1, S8). Strategies were allocated to an analytic category of “no intention,” “doing,” “considering,” or “stopped” (Table 2). In this article, findings are described across two dimensions: likelihood and commonness. Regarding likelihood, this article discusses the strategies most likely to be allocated to one of the four analytic categories. Regarding commonness, this article discusses the responses that were most common for a strategy. It should therefore be noted that the most common response for an individual strategy may differ from the analytic category that it was most likely to illustrate. For example, of all 16 strategies, S2 was the most likely to be stopped (22.0%) but the most common response for this strategy was “no intention” (31.4%).

As the aims of this article are deductive, open-text comments from surveys and discussions generated during exit interviews were not subject to inductive qualitative data analysis. However, some detail from open-text survey questions and semi-structured exit interviews have been included to supplement, illustrate and expand upon the survey data findings where relevant.

## Findings

### Participants

Seventeen people participated in the study (12 female, 5 male) representing 14 thoroughbred breeding farms and three equine veterinary centers/practices. They identified their roles as stud farm management (8), equine veterinarian (6), equine nurse (2) and stud farm staff (1). In this article, participants are referred to as “Px.”

For the 14 stud farms represented in the study, the total number of full-time equivalent (FTE) staff during the 2021 foaling season was estimated at 134, ranging from two to 22, with an average of 10 and a median of seven staff. Estimates included night watch and day shift staff. The total number of mares foaled down at the 14 stud farms was 1,777, ranging from 25 to 337 with an average of 127, and a median of 89 mares.

For the three veterinary business represented in the study, the total number of FTE staff (veterinarians and veterinary nurses) employed during the 2021 foaling season was 26, ranging from one to 13 with an average of nine and a median of 12. The total number of mares foaled by the three veterinary businesses was 212, ranging from 30 to 122, with an average of 71 and a median of 60 mares.

### Zoonoses most relevant to participants

As noted above, participants were asked what in their opinion were the top three zoonoses about which staff should be aware during routine equine reproduction work. This was a free text question in the first self-audit survey. Nine different zoonotic diseases were noted by the 17 participants. One participant also listed equine influenza, which was later excluded as it is not transmissible to humans (although it may have zoonotic origins). The top three zoonoses were, in descending order of mentions: chlamydiosis, salmonellosis and HeV. The full range and count of responses is shown in Table 3.

**Table 3:** The top three zoonoses about which participants thought they should be aware during routine equine reproduction work.

**Zoonotic disease**	**Mentions**
Chlamydiosis (*Chlamydia psittaci*)	13
*Salmonellosis*	12
Hendra Virus (HeV)	10
*Cryptosporidiosis*	6
*Leptospirosis*	3
Australian bat lyssavirus (ABLV)	1
*Escherichia coli*	1
*Dermatophilosis*	1
Methicillin-resistant *Staphylococcus aureus* (MRSA)	1

One participant used the “any other comments” box to elaborate on their response to this question, as follows:

Primary concern is Hendra virus in any unvaccinated animal which presents with potential symptoms. Next level of concern is hygiene in diarrhea cases which may be *cryptosporidia* or *salmonella*. Final level of concern is hygiene with placentitis mares at foaling and handling of suspect placentas post-foaling (P3, W1).

### Stage of adoption reported across all waves

**Figure 1:**
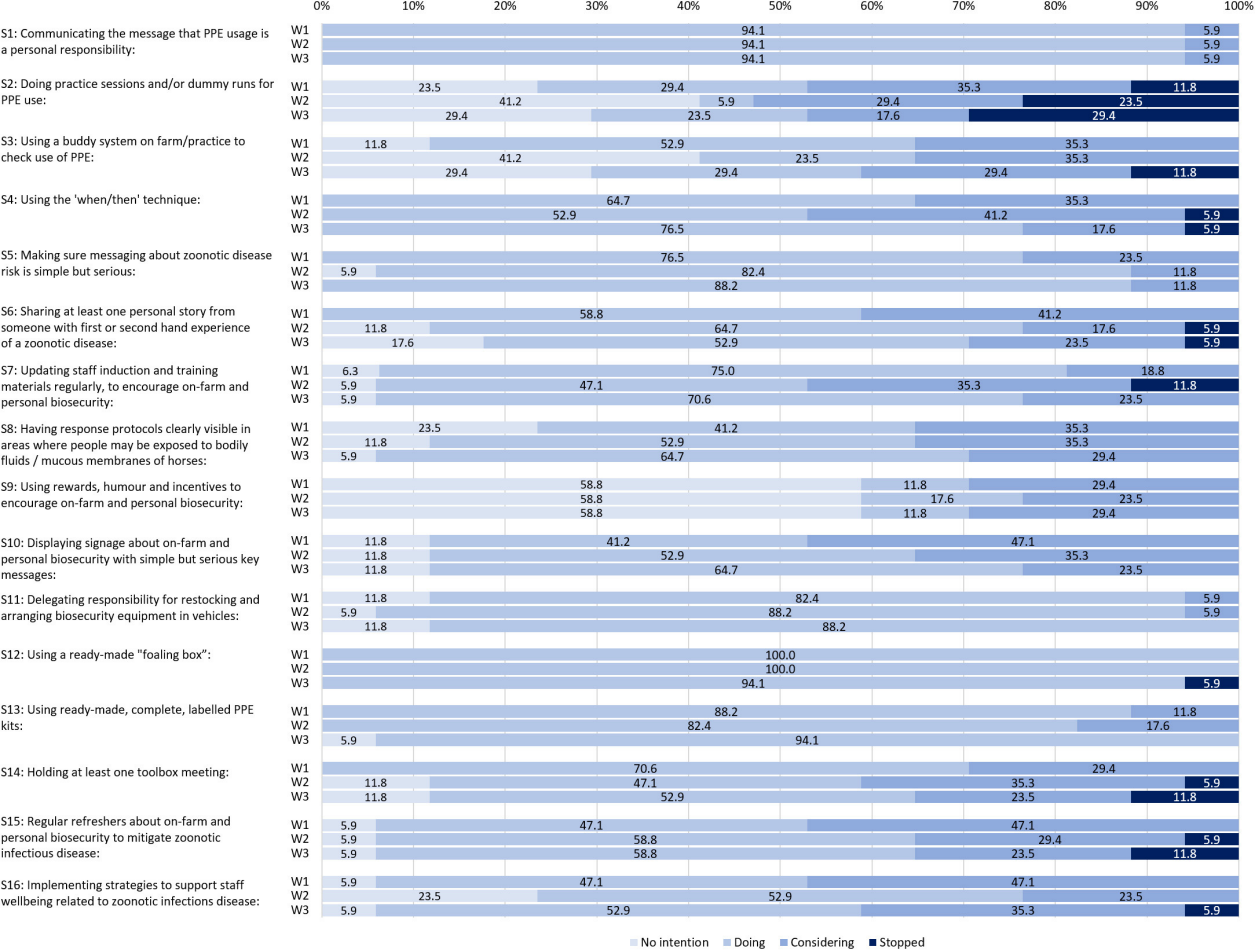
The percentage of participants at different stages of strategy adoption, disaggregated across three self-audit survey waves.

The percentage of participants at each of the four TTM-inspired stages of change across the three self-audit survey waves is illustrated in Figure 1.

**Figure 2:**
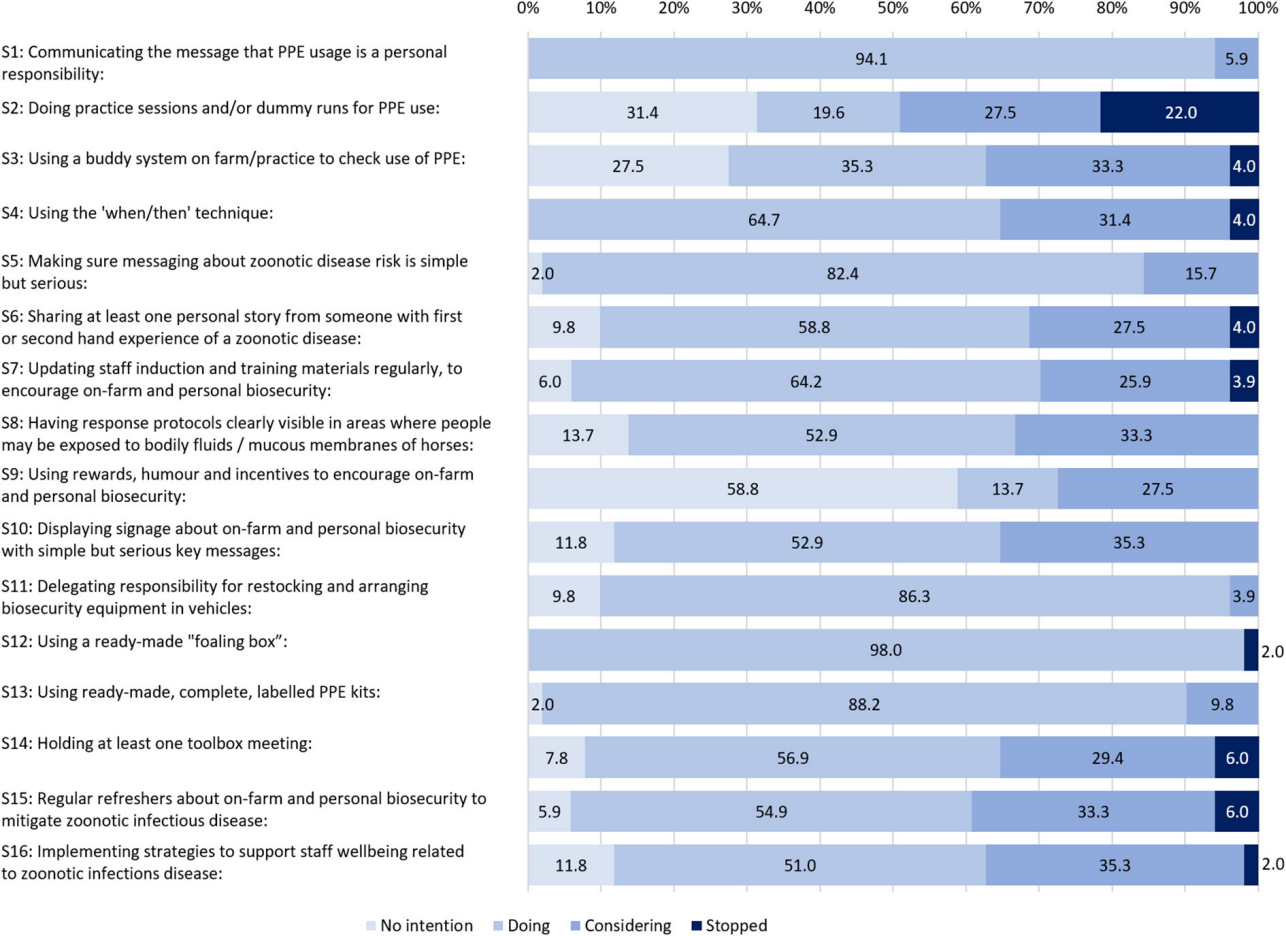
The percentage of participants at different stages of strategy adoption with all three waves aggregated.

To identify overall trends for strategy adoption, findings for the three self-audit survey waves were aggregated (see Figure 2).

### No intention: Strategies that participants were least likely to adopt

The strategy that participants were most likely to report as having “no intention” to adopt was S9: “using rewards, humor and incentives to encourage on-farm and personal biosecurity” (Figure 2), which was consistently 58.8% at each data collection wave (Figure 1). The other two strategies most likely to have “no intention” were:

- S2 “doing practice sessions and/or dummy runs for PPE use” (31.4%), and
- S3 “using a buddy system on farm/practice to check use of PPE” (27.5%) (Figure 2).

S9 was discussed with participants during exit interviews. Many were confused by the mixture of rewards, humor and incentives in the wording of one strategy. Many participants used, valued and encouraged the use of humor in their workplaces to emphasize biosecurity messages. However, they disagreed with the premise of rewarding or incentivising personal biosecurity. They felt that personal biosecurity was not optional and as such did not need to be incentivized or rewarded. In other words, personal biosecurity at many stud farms and veterinary practices was expected. Participants also struggled to imagine how they would incentivise or reward personal biosecurity, apart from the inherent benefit of not getting sick. Where they supported humor but disagreed with the premise of the strategy, participants appeared to err on the side of choosing the “no intention” survey response.

Survey comments and exit-interview discussions revealed that S2 and S3 were both considered more relevant and easier to undertake prior to foaling commencing. Both strategies were also considered more relevant for new and novice workers. As explained by one participant during a computer-assisted telephone interview to complete their W2 survey:

Buddy system not so relevant because they are all experienced. It is more so only relevant for newbies. (P14).

One participant's survey responses for all waves were almost exclusively “we already do this” or “we've no intention of doing this.” Exit interview discussion revealed that many strategies were considered irrelevant for this smaller operator, for two reasons. First, a high workload spread amongst a small team made it hard to allocate time to activities perceived as “additional” to core foaling tasks. Second, the intimacy of a small team made some strategies irrelevant, such as “holding a toolbox meeting.” For example,

Please note our farm is [only a few people] (and some backpackers) so we don't hang signs or have tool box talks. We obviously discuss these things but in a non-formal environment (P11, W1).

Other reasons for having no intention of adopting a strategy related to seeing no need. This perception was mostly described by participants in terms of worker experience, as has been noted above in relation to experienced staff being confident in their ability to don and doff PPE without needing a PPE buddy. However, one participant felt there was no need to implement many of the strategies because the farm had implemented more primary controls over biosecurity hazards. They described a situation where it was so difficult to secure skilled staff who could catch and handle horses that most staff were restricted from any kind of interaction with horses. This meant that two skilled members of staff managed almost all the work involving direct interactions with horses.

### Doing: Strategies that participants were already likely to be doing

As shown in Figure 2, the top three strategies that were being done across all waves were:

- S12 “using a ready-made foaling box” (98.0%),
- S1 “communicating the message that PPE usage is a personal responsibility” (94.1%), and
- S13 “using ready-made, complete, labeled PPE kits” (88.2%).

S1 had the same high rate of adoption (94.1%) for each self-audit survey wave and was the only strategy for which there was a consistent adoption rate across all survey waves (Figure 1).

In W1, all sixteen strategies were being practiced by at least one participant and ten strategies were being practiced by at least 50% of participants. Across the foaling season, all but S2, S3, and S9 were in use by at least 40% of participants at each wave (Figure 1).

During exit interviews, participants were asked if they thought that the use of PPE such as masks and gloves had increased from the previous foaling season. All believed this to be the case, something that they attributed to the COVID-19 pandemic and broader Public Health directives in Australia, specifically around the wearing of masks. However, some open-text survey comments about PPE-use revealed varying opinions about when it was required:

Making sure all staff use appropriate PPE provided when in contact with bodily fluids that is foaling and repro work (P14, W1, “already do this”).

Regular use of PPE for any suspected infection cases (P4, W2, “still doing this”).

Still, participants noted areas for improvement. For instance,

There are entrenched practices of stud farms not using PPE when it is applicable so it is baby steps as far as discussing better ways to do things so far (P9, W2).

Ten other strategies related to PPE use were described by participants in the self-audit survey, under the optional survey question for “something else” (Table 4). Eight were already being practiced and two were being considered or planned.

**Table 4:** Ten other strategies related to personal protective equipment (PPE) that were described by participants in the self-audit survey, under the optional survey question for “something else”, showing status as “doing,” or “considering”.

**“Something else”: Any other strategy reported by participants in the self-audit survey**	**Status**
1. Using high pressure cleaners for crushes and boxes	Doing
2. Using single use foaling kits	Doing
3. Staff reporting any potential zoonotic symptoms	Doing
4. Cleaning and disinfecting crushes and boxes	Doing
5. Putting assorted sized gloves in display racks	Doing
6. Using foot baths	Doing
7. Having hand sanitizer at every crush, and	Doing
8. Putting blood Immunoglobulin G tubes in sharps container (not bin)	Doing
9. An online zoonoses learning module	Considering
10. Preparing an easy to use guide relating to zoonoses and personal protective equipment (PPE), that would be disseminated to vets going out to stud farms, intended to inform vets and promote discussion with client stud farms	Considering

As noted above, W1 contained slightly different options to distinguish between strategies that had been adopted prior to study participation, but not before the stakeholder workshop (when strategies were suggested by industry stakeholders). This enabled the identification of 13 strategies that were adopted by at least one participant following participation in the workshop and prior to the study commencing (Table 5). Of those 13 strategies, four were adopted by more than one participant following participation in the workshop and prior to the study commencing: S3 “using a buddy system on farm/practice to check use of PPE,” three started S7 “updating staff induction and training materials regularly, to encourage on-farm and personal biosecurity,” three started S4 “using the ‘when/then' technique to change behaviors,” and two started S11 “delegating responsibility for restocking and arranging biosecurity equipment in vehicles.”

**Table 5:** The 16 strategies already in use at the first survey wave (W1), showing strategies adopted before and after the workshop on 1 June, 2021, ranked from highest to lowest number of participants reporting their usage.

**Strategy**	**Already doing this before workshop on 1 June, 2021**	**Started doing this after workshop on 1 June 2021**	**Total number of participants already using the strategy at W1**
S12: Using a ready-made “foaling box”	17 (100.0%)	0 (0.0%)	17 (100.0%)
S1: Communicating the message that personal protective equipment (PPE) usage is a personal responsibility that needs to be self-disciplined/motivated	15 (88.2%)	1 (5.9%)	16 (94.1%)
S13: Using ready-made, complete, labeled PPE kits	14 (82.4%)	1 (5.9%)	15 (88.2%)
S11: Delegating responsibility for restocking and arranging biosecurity equipment in vehicles	12 (70.6%)	2 (11.8%)	14 (82.4%)
S5: Making sure messaging about zoonotic disease risk is simple but serious	12 (70.6%)	1 (5.9%)	13 (76.5%)
S7: Updating staff induction and training materials regularly, to encourage on-farm and personal biosecurity (*n* = 16)	9 (56.3%)	3 (18.8%)	12 (75.0%)
S14: Holding at least one toolbox meeting	11 (64.7%)	1 (5.9%)	12 (70.6%)
S4: Using the “when/then” technique to change behaviors	8 (47.1%)	3 (17.6%)	11 (64.7%)
S6: Sharing at least one personal story from someone with first or second hand experience of a zoonotic disease with staff on my farm/practice	10 (58.8%)	0 (0.0%)	10 (58.8%)
S3: Using a buddy system on farm/practice to check use of PPE	5 (29.4%)	4 (23.5%)	9 (52.9%)
S15: Regular refreshers about on-farm and personal biosecurity to mitigate zoonotic infectious disease	7 (41.2%)	1 (5.9%)	8 (47.1%)
S16: Implementing strategies to support staff wellbeing related to zoonotic infectious disease	7 (41.2%)	1 (5.9%)	8 (47.1%)
S8: Having response protocols clearly visible in areas where people may be exposed to bodily fluids/mucous membranes of horses	6 (35.3%)	1 (5.9%)	7 (41.2%)
S10: Displaying signage about on-farm and personal biosecurity with simple but serious key messages	6 (35.3%)	1 (5.9%)	7 (41.2%)
S2: Doing practice sessions and/or dummy runs for PPE use	4 (23.5%)	1 (5.9%)	5 (29.4%)
S9: Using rewards, humor and incentives to encourage on-farm and personal biosecurity	1 (5.9%)	1 (5.9%)	2 (11.8%)

### Considering: Strategies being considered or adopted

For all survey waves combined (Figure 2), the top five strategies most likely to be under consideration, but not yet adopted, were:

- S10 “displaying signage about on-farm and personal biosecurity with simple but serious key messages” (35.3%),
- S16 “implementing strategies to support staff well-being related to zoonotic infectious disease” (35.3%),
- S3 “Using a buddy system on farm/practice to check use of PPE” (33.3%),
- S8 “Having response protocols clearly visible in areas where people may be exposed to bodily fluids / mucous membranes of horses” (33.3%), and
- S15 “regular refreshers about on-farm and personal biosecurity to mitigate zoonotic infectious disease” (33.3%).

Regarding S10 “displaying signage about on-farm and personal biosecurity with simple but serious key messages,” the following comments were all made in response to the W2 survey:

…we have taken some posters from the HTBA [Hunter Thoroughbred Breeders Association] website^2^ and printed them out and laminated them for placement around the farm for more awareness (P2).

The busy breeding season makes it difficult to change habits right now but there are ways to build this going forward using graphic and written resources (P9).

Assorted laminated biosecurity posters on display (P4).

Two strategies stood out for showing no consistent trend toward or away from adoption. These were S2 “Doing practice sessions and/or dummy runs for PPE use” and to a lesser extent, S3 “Using a buddy system on farm/practice to check use of PPE,” both from Theme 1 “Encouraging appropriate PPE use.” For S2, all four stages of change were indicated by at least 19.2% of participants on aggregate and for S3, three stages of change were indicated by at least 27.5% of participants on aggregate (Figure 2). These same two strategies also featured amongst the three strategies that participants were most likely to report having no intention to adopt (S2, S3, S9).

### Stopped: Strategies that were most likely to be discontinued

The single strategy most likely to have been “stopped” was S2 “doing practice sessions and/or dummy runs for PPE use” (22.0%). Other strategies most likely to be stopped had a much lower percentage, with 6.0% (S14 and S15) or 4.0% (S3-4, S6-7). Comments in the survey tool revealed how the demands of foaling made S2 difficult to arrange. For example,

They were doing dummy runs but stopped due to breeding season (P14, W2, CATI with researcher).

Another participant emphasized the difficulty of incorporating personal biosecurity strategies into the work demands of foaling more generally:

We have stopped doing some things because we are so busy in the season – will restart next year probably (P17, W2).

Nine of the 16 strategies were “stopped” at some point in the self-audit survey waves, including in W1 (Figure 1). Seven strategies were never reported as having been stopped at any wave S1, S5, S8–11, and S13 (Figure 2).

## Discussion

### Awareness of zoonoses

Together, participants felt that a range of zoonotic diseases were relevant to their work. Findings suggest that *C. psittaci, Salmonellosis* and HeV are most likely to resonate with this group. These findings are not unexpected. A 2014 outbreak of *C. psittaci* (11) led to the serostudy that was a precursor to the present study (13) and which involved some of the participants. A review of samples from 600 aborted foals across Australia over a 25-year period (1994–2019), identified *C. psittaci* in 3.9% of samples from New South Wales (36), compared with an overall prevalence of 6.5%. Exit interviews suggested that *Salmonellosis* was a routine concern for workers. Prevalence in Australia is unclear, with most research focused on identifying strains in veterinary hospitals (37, 38). In relation to future educational or behavior change interventions, these three zoonoses considered most relevant could serve as particularly engaging examples for broader information and communication about equine zoonotic diseases. At the same time, awareness about other zoonoses relevant to the equine industry such as methicillin-resistant *S. aureus* (MRSA) infection may need to be increased, especially given that “nasal MRSA colonization of veterinarians and veterinary personnel is frequent in horse clinics” [(39), p. 63].

An equine HeV case in the greater Newcastle area in October 2021 (40) did not impact responses, as it occurred after all surveys had been completed for the first wave of the self-audit survey. However, the inclusion of HeV amongst responses to the “top three” zoonoses question may have been impacted by a case about 2 years prior in the Hunter region (41). As a potentially fatal zoonosis, HeV likely resonated highly with participants due to the seriousness of its consequences.

### The rejection, adoption, and cessation of personal biosecurity strategies

Participants frequently justified their selection of a “no intention” or “stopped” response on the basis that foaling had ended at their workplace and that the strategy was therefore no longer necessary. Thus, a response of “stopped” in many cases reflected the cessation of the perceived need for that strategy. Still, only one strategy out of the 16 in this study was worded specifically around foaling; S12 “using a ready-made foaling box.” This may suggest that many of the strategies that were relevant to general biosecurity practices were only considered by participants to be relevant in relation to foaling-specific practices, at least for the purposes of completing the survey. Whilst the introductory text to each wave did not specifically mention foaling, the construction of the study as one being undertaken over the 2021 foaling season may have biased participants to respond from the perspective of foaling-related biosecurity risks and practices. Still, whilst certain known zoonoses can be classified as high/low likelihood and high/low consequence during particular times of the year or specific kinds of interactions with horses, the likelihood and consequence of novel and emerging zoonoses from any interaction is, of course, unknown (42).

The self-audit survey data suggest that personal biosecurity strategies were widely and variously used by participants in this study. This could be considered a favorable level of personal biosecurity, although there is no comparative data from previous seasons against which to compare our findings. By raising broad awareness of biosecurity (43), the COVID-19 pandemic may have normalized the use of masks and other related PPE such as gloves - although a study of veterinary workers in Washington state, USA found only a slight increase in PPE use when comparing behavior before and during the pandemic (44). Furthermore, there are no industry standards. If the ideal for personal biosecurity strategies was 100% adoption, 100% of the time, our findings identify a need for further reinforcement and promotion.

A particularly encouraging finding can be inferred from the one strategy that was strikingly unpopular; S9 “using rewards, humor and incentives.” In the literature on organizational safety, the functional capacity of culture is often described simply as “the way we do things around here” [(45), p. 21]. The finding that participants disagreed with the premise of incentivizing or rewarding personal biosecurity precisely because it was expected suggests that personal biosecurity has become expected and normalized within the workplace culture of the thoroughbred breeding industry in the Hunter Valley. That is, biosecurity was part of the way things were done in most of the workplaces included in this study, most of the time. As exit interviews revealed widespread support for the use of humor, further surveys or interventions should distinguish and track humor as a separate strategy from “incentives and rewards.”

Strategies that for at least one wave were most likely to be in consideration but not adopted were S10 “displaying signage about on-farm and personal biosecurity with simple but serious key messages,” S15 “regular refreshers about on-farm and personal biosecurity to mitigate zoonotic infectious disease” and S16 “implementing strategies to support staff well-being related to zoonotic infectious disease,” although little was discovered about what specific strategies were in use. In terms of behavior change, these are the strategies that could be expected to move to the adoption stage if targeted with further intervention.

Few strategies were stopped during the study, but those that were, were simply deemed irrelevant due to foaling having already commenced or having slowed or stopped, or due to the time-pressures of foaling. The strategy of “doing practice sessions and/or dummy runs for PPE use” seemed to be particularly difficult for participants to maintain during the foaling season. This particular strategy may enjoy more widespread adoption and maintenance if it was encouraged prior to the foaling season. Alternatively, this may be a strategy requiring particular encouragement during the foaling season, alongside strategies or support to make it achievable.

Findings demonstrate the impact of the wording of strategies on responses. The inclusion of “humor,” “incentives” and “rewards” in S9 has already been discussed. There was also a mixture of both specific and vaguely-worded strategies that could be reconsidered, sometimes within the same strategy. For example, S14 refers specifically to a “toolbox meeting” which may resonate with larger, formalized operations but not with small family operations where the intended principle of frequent communication updates still occurs. At the same time, S14 refers to holding at least one meeting which were not quantified as daily, weekly, monthly or per foaling season.

Above, we discussed the case of a site struggling to secure skilled horse handlers where the use of specific biosecurity strategies was effectively trumped by restricting access to horses. From the perspective of the hierarchy of controls, this was an attempt to eliminate the hazard (horses and horse material) (46). This workaround was adopted out of necessity and reflected a shortage of skilled workers capable of handling horses safely. Whilst tools from the framework of workplace health and safety have much value in equestrian industries (47), this strategy introduced a vulnerability to zoonotic disease prevention by concentrating responsibility and risk to one or two staff members who were already experiencing the heightened workload of the foaling season and who – in an era of COVID-19, could have been required to self-isolate. Moreover, such a strategy relies on full compliance by workers who may be too unskilled to even recognize when they are coming into direct contact with hazardous material.

Importantly, when asked in exit interviews if there was anything they had tried in the 2021 season that would not be tried again in the 2022 seasons, all participants indicated they would be willing to repeat strategies they had tried.

### Significance, limitations, and further directions

As noted above, the TTM was developed to understand and motivate human behavior change for human health. In a One Health context such as zoonotic disease prevention, human behavior change makes sense in relation to and can be motivated by, human and animal health. The application of the TTM beyond human health behaviors is still a nascent field characterized by a relatively *ad hoc* application of one or more of the four core TTM constructs (stages of change, self-efficacy, decisional balance and processes of change) variously to study design and/or the interpretation of findings. Regarding studies of horse welfare, the authors of an Australian study of HeV observed how the TTM stages of behavior change could be discerned in the management behavior of veterinarians and the government (48), whilst the authors of an interview-based study of laminitis considered their findings about how farriers communicate to horse owners comparable with the TTM (49). Even without considerable empirical evidence for its application to human behavior change for animal welfare, the TTM has been recommended to veterinarians as a suitable device for assessing pet owners' readiness to implement a weight management program for their pets (50). However, these studies have not systematically utilized all four core TTM constructs across research design, data collection and interpretation of findings. Still, one study of dairy cattle farmers' biosecurity attitudes and behaviors in Great Britain employed a TTM-influenced survey design similar to that described in the present study (51). Whilst the present study did not utilize all TTM constructs, the TTM stages of change construct was used to systematically structure data collection, presentation and interpretation. To our knowledge, this is the first study to use the TTM for the purposes of understanding and monitoring human behavior change for both human health and equine welfare. The present study is also relatively novel for contributing a solutions-focused approach to a field of research which has necessarily been concerned with characterizing “the problem” of zoonoses and describing barriers to the uptake of recommended mitigation strategies (17, 23).

Criticisms of the TTM question its predictive power, implied linearity of change and artificially discrete stages (52, 53). However, in the present study, the TTM provided a useful device for identifying adoption rates of biosecurity strategies over the 2021 thoroughbred breeding season, with some interesting reflections for future application to behavior change around zoonotic disease prevention.

First, whilst researchers acknowledge that some addictive behaviors like smoking and drinking are more difficult to change at certain times of the year, such as Christmas and New Year celebrations, the TTM was developed to identify and address behaviors with continual relevance. In contrast, the focus of our study was on the foaling season when people working with horses were more likely to come into contact with biological materials that could transmit zoonoses. As our study targeted a seasonal context (foaling), “stopped” responses in self-audit survey data should not be interpreted as regression or relapse but more as evidence of a relevance judgment made by participants. In other words, where a strategy is described by a participant as “stopped,” this may more accurately reflect the cessation of the activity thought to necessitate those behaviors (i.e., foaling).

Second, the TTM was developed to understand and change behaviors that are cumulatively dangerous for human health, like smoking, unhealthy food choices and inactivity (54). The likelihood and severity of negative health effects from engaging in these activities increases over time. However, in relation to preventing zoonotic disease, single exposures can lead to zoonotic transmission, the effects of which can be devastating. Therefore, whilst the overall findings of the present study provide an encouraging picture of willingness to adopt a variety of on-farm and personal biosecurity practices in the Hunter thoroughbred breeding industry over the 2021 breeding season, adoption rates fell short of 100% compliance, 100% of the time. As noted by Weese, “the preventable fraction for equine infectious diseases is completely unclear but, certainly, a reasonable percentage of equine infections could be prevented through application of basic infection control measures” [(18), p. 658]. Data are not available for non-notifiable equine zoonotic infection amongst humans to provide any comparative evidence for the relative benefits of each or any of the strategies that were included in the study. Still, there is a need for industry-wide discussions about what levels of risk are and are not acceptable. Agreement will be complicated by potentially competing demands from worker health and safety and public health frameworks within which specific horse-human interactions occur, in locations subject to their own local, regional and industrial cultures and uncontrollable environmental conditions.

Whilst we were able to categorize readiness for change in four ways based on the TTM stages of change construct, our self-report survey methodology was oriented toward the collection of stated behaviors. As a result, there is still a need to conduct observational research with thoroughbred studs and veterinary centers to document revealed behaviors and triangulate them with self-report findings. One potential benefit of observational research could be the identification of informal, site-specific strategies that could be considered for broader promotion, as has occurred in other high risk/low occurrence organizational contexts such as firefighting (55).

Moreover, our behavior-based study did not collect data on the important psychological, attitudinal or cultural dimensions of biosecurity in the thoroughbred breeding industry. This could be achieved by involving two other constructs from the TTM, notably decisional balance and self-efficacy. However, there is also significant potential benefits in pairing the TTM with a complimentary theory of behavior change. For example, authors of the aforementioned study of dairy cattle farmers' biosecurity measures (51) paired the TTM with Aizen's Theory of Planned Behavior (TPB) (56). In that study, the TPB provided a structured means to identify and takes into consideration the role of farmers' attitudes, subjective norms and perceived control on their uptake of biosecurity measures. By combining the TTM with the TPB, the researchers report being able to “address both the motivation (TPB) and action (TTM) aspects of individual farmer behavior change…” This combination of the TTM and the TPB has been advocated for another area where human health and animal welfare are intertwined; antimicrobial resistance (57). For the present concern of zoonotic disease prevention via personal biosecurity strategy adoption, the TPB could help to reveal workers' biosecurity priorities, attitudes and perceptions of control. Thus, further research based on the TPB and including other TTM constructs such as self-efficacy could provide a more thorough understanding of the broader socio-cultural context within which biosecurity practices are made meaningful by workers in the thoroughbred industry. Such in-depth research into personal biosecurity and health would augment existing research on safety in human-horse interactions which has been biased toward accident and injury (3, 58), as noted earlier.

The findings of this study should be considered in relation to the identification, self-auditing and wording of strategies. The 16 strategies that were included in study were proposed by industry participants at a stakeholder workshop some 3 months prior to data collection. These strategies were generated from an organized interaction between researchers and industry stakeholders as well as industry champions and co-researchers who represented research and industry. This co-production of strategies was a result of genuine collaboration with stakeholders which most likely contributed to ongoing engagement with researchers. However, all strategies were assumed to have a positive and equal impact on improving personal biosecurity and reducing zoonotic disease infection. Whilst this assumption is not unreasonable, “there are limited objective data regarding the usefulness of most infection control measures in horses, including longstanding and widely used practices” [(18), p. 658].

There are also several important considerations regarding the self-auditing of strategies, which may have been subject to a social desirability bias whereby participants may have exaggerated their adoption of strategies (59). However, the willingness with which participants appeared to select “not intended” or “stopped” suggests that this tendency may have been weak. The fact that all strategies were worded positively around the uptake of behaviors (rather than the cessation of negative ones) may also have mitigated against participants appeasing researchers with what they perceived to be the most desirable responses. It is also worth noting that whilst tick box responses were convenient for data collection and analysis, failing to include a “not applicable” option may have compromised validity. Indeed, when participants were presented with printouts of their responses from the survey waves in the exit interviews, several commented that they may have “selected the wrong thing.” Further discussions in exit interviews suggested that in many instances, participants would have selected “not applicable” if it had been provided as a response option.

This study was completed by a small, non-representative sample; 17 participants from 14 thoroughbred breeding farms and three equine veterinary centers. Most of the organizations reflected in the sample were medium to large operators, capable of committing staff time to multiple survey waves and exit interviews. The modest sample size and bias toward large operators reflects the detailed methodology requiring personal contact with researchers and industry co-researchers. Further research with a statistically significant sample size is required to contextualize findings in relation to other thoroughbred breeding farms in the Hunter Valley, other States/Territories and the national thoroughbred breeding industry – as well as other types of horse breeders such as the Standardbred breeding industry, other popular horse breeds in Australia (e.g., Australian stock horses, warmbloods, colored horses), and hobby and “one-off” breeders.

In particular, a national survey would make it possible to determine personal biosecurity strategy adoption (i.e., behaviors), as well as attitudes, norms and perceived control. It would also enable comparisons according to knowledge, training, type of employment (i.e., full-time, part-time, casual, seasonal), type of horse/equine sector, and role (e.g., veterinarian, stud manager, foaling attendant, veterinary nurse, stud hand, owner-breeder). The latter is especially important given only three veterinarians participated in the present study. Whilst veterinarians are more likely to interact with “high risk” sick or problematic horses in purpose-designed treatment spaces, many also work on farms where they are involved in routine, “low risk” foaling. Indeed, many of the (larger) studs have their own resident veterinarians who are part of the farm management team, and often reside on farm.

The need to include small operators in future research will also be valuable, given that in Australia, “[m]ore than half of all mares are owned by breeders with five or fewer horses.”^3^ Moreover, whilst the risk of zoonotic disease infection is heightened in and around foaling, there are other aspects of the thoroughbred breeding industry deserving of further biosecurity research and extension. These include stallion barns, yearling barns, sales yards, transport and race preparation.

Finally, zoonotic diseases do not discriminate by pedigree! Whilst the thoroughbred industry is governed by well-respected and enforced vaccination regimes (e.g., HeV), there is a need to determine and evaluate the attitudes and behaviors of breeders of other types of horses as well as “backyard,” once-off and leisure horse breeders. This is particularly important in light of unknown and emerging zoonotic diseases for which there are no vaccinations or recognized indications for vaccination (42, 60). In fact, one study of emerging infectious diseases that occurred between 1940 and 2004 found that over 60% were zoonotic (61).

Notwithstanding these limitations, the present study suggests that the TTM – alone or in combination with other complementary behavior change theories such as the TPB, can provide a useful methodological framework for trialing, monitoring and encouraging strategies for zoonotic disease prevention in other equine contexts and animal-related industries in Australia or overseas. Regardless of the theoretical and methodological design, future research intended to reduce the risk of zoonotic disease infection to horses and humans will have the greatest chance of success when undertaken within a One Health framework (62, 63) involving multidisciplinary research teams, as occurred with the present study.

## Conclusion

This multi-site study of 16 personal biosecurity strategies in the Hunter Valley thoroughbred breeding region suggests that there is a willingness for workers to adopt and maintain practices designed to reduce the risk of zoonotic disease infection and transmission. However, there is capacity for more strategies to be implemented more often. Findings suggested that future interventions designed to increase the uptake of personal biosecurity strategies will need to be sensitive to the time of the year, the size of the operation and the skills of workers. Specifically, they should be designed to assist stakeholders in the thoroughbred breeding industry to (a) identify which kinds of strategies require what kind of support to be adopted and normalized in their specific workplace, and (b) determine optimal timing for messaging to raise awareness, encourage uptake of personal biosecurity strategies and support their maintenance to reduce the risk of zoonotic disease.

## Data availability statement

The raw data supporting the conclusions of this article will be made available by the authors, without undue reservation.

## Ethics statement

This study involving human participants was reviewed and approved by Hunter New England Human Research Ethics Committee (2019/ETH01070). The participants provided their written informed consent to participate in this study.

## Author contributions

KT conducted exit interviews, analyzed self-audit survey data, and drafted the manuscript. JT, CC, and JC assisted with participant recruitment. CC and JC liaised with participants, facilitated rapport with the research team, and provided specialty support and subject matter expertise. DM created videos and other downloadable resource materials to support participants and extend earlier research findings on which the current work is based. All authors contributed to the design of the methodology, design of data collection tools, interpretation of findings and editing of the final manuscript, and share accountability for the content of this article.

## Footnotes

^1^REDCap (Research Electronic Data Capture) is a secure, web-based application designed to support data capture for research studies, providing 1) an intuitive interface for validated data entry; 2) audit trails for tracking data manipulation and export procedures; 3) automated export procedures for seamless data downloads to common statistical packages; and 4) procedures for importing data from external sources.

^2^ https://www.htba.com.au/biosecurity

^3^ https://www.tbaus.com/australian-breeding/our-industry
